# Efficacy and safety of milrinone infusion in patients with aneurysmal subarachnoid hemorrhage: a systematic review and meta-analysis

**DOI:** 10.3389/fneur.2026.1872442

**Published:** 2026-09-09

**Authors:** Khaled Gharaibeh, Aizaz Ali, Nameer Aladamat, Ashfaq Shuaib, Syed F. Zaidi, Mouhammad A. Jumaa

**Affiliations:** 1 College of Medicine and Life Sciences, University of Toledo, Toledo, OH, United States; 2 University of Alberta, Edmonton, AB, Canada

**Keywords:** delayed cerebral ischaemia, milrinone, outcome, subarachanoid haemorrhage, vasospam

## Abstract

**Background:**

Aneurysmal subarachnoid hemorrhage (aSAH) is a life-threatening neurological condition that can be complicated by delayed cerebral ischemia (DCI), a leading cause of secondary brain injury. Milrinone, a phosphodiesterase-3 inhibitor with vasodilatory and anti-inflammatory properties, has been proposed as a potential therapeutic agent to mitigate DCI, yet its clinical utility remains uncertain. This systematic review and meta-analysis aimed to evaluate the efficacy and safety of milrinone infusion in patients with aSAH.

**Methods:**

We systematically searched PubMed, Embase, and Cochrane Library databases from inception through June 1, 2025. Eligible studies included randomized and observational trials comparing outcomes between patients treated with and without milrinone infusion following aSAH. This analysis used aggregate study-level data; individual patient–level data were not available. Primary outcomes included incidence of DCI, good functional outcome (modified Rankin Scale score 0–2), and mortality. Cardiovascular adverse events were also assessed. Data were pooled using a random-effects model, and heterogeneity was evaluated using the *I*^2^ statistic.

**Results:**

Seven studies comprising 1,045 patients across all routes of milrinone administration were included, of whom 529 (50.6%) received milrinone. In the primary analysis restricted to intravenous milrinone, treatment was associated with lower odds of vasospasm-related DCI (OR 0.45, 95% CI 0.23–0.90; *p* = 0.02). However, this association was not robust to leave-one-out sensitivity analysis. Intravenous milrinone was not associated with significantly better functional outcomes (OR 1.47, 95% CI 0.84–2.56; *p* = 0.17) or reduced mortality (OR 0.95, 95% CI 0.48–1.88; *p* = 0.88). Cardiovascular adverse events were numerically more frequent with intravenous milrinone, although the difference was not statistically significant (OR 1.63, 95% CI 0.86–3.08; *p* = 0.13).

**Conclusion:**

Milrinone administration in patients with aSAH is associated with a lower incidence of delayed cerebral ischemia, with a trend toward increased cardiovascular adverse events. However, this did not translate into improved functional outcomes or reduced mortality. Further large-scale randomized controlled trials are needed.

## Introduction

Aneurysmal subarachnoid hemorrhage (aSAH) remains a life-threatening neurological emergency with significant long-term morbidity and mortality (1). Among the complications that contribute to poor outcomes in aSAH, delayed cerebral ischemia (DCI) is recognized as a leading cause of secondary brain injury, affecting up to 30% of patients and contributing to functional decline and cerebral infarction despite successful aneurysm repair (2). DCI after aSAH is a multifactorial process extending beyond large-vessel vasospasm. Elevated von Willebrand factor and relative ADAMTS-13 deficiency promote platelet-rich microthrombi, while inflammation-mediated glycocalyx degradation further facilitates platelet adhesion and microvascular dysfunction. Concurrent AQP4-dependent glymphatic impairment reduces the clearance of blood products, metabolic waste, and damage-associated molecular patterns, thereby amplifying neuroinflammation and cerebral hypoperfusion (3).

Milrinone, a phosphodiesterase-3 inhibitor, has been increasingly used in this setting due to its combined inotropic and vasodilatory properties (4). These mechanisms offer a clinical approach to mitigating both macrovascular vasospasm and microcirculatory impairment, providing the rationale for its use in DCI management. Despite growing interest in milrinone and its potential to reduce the incidence of vasospasm-related cerebral infarction and the need for endovascular rescue therapy, the evidence base remains limited to small single-center studies with heterogeneous protocols and outcome measures.

Although several narrative reviews have summarized the clinical use of milrinone in DCI, no prior study has quantitatively synthesized the available data through meta-analysis. By performing both a systematic review and meta-analysis, this study provides pooled estimates of milrinone’s efficacy and safety, offering numerical evidence to support its clinical use. This approach addresses a critical knowledge gap, clarifies milrinone’s role in contemporary neurocritical care, and lays the groundwork for future randomized controlled trials.

## Methods

We conducted this systematic review and meta-analysis based on the guidelines of the Preferred Reporting Items for Systematic Reviews and Meta-analysis (PRISMA) (5). Data is available on request to the corresponding authors.

### Data source and search strategy

We searched PubMed, Embase and Cochrane Library to obtain articles in all languages from inception until June 1, 2025. “Subarachnoid Hemorrhage” OR “SAH,” “vasospasm” OR “delayed cerebral ischemia,” and “milrinone” were the search terms. Synonyms were obtained from PubMed and Embase with elimination of duplicates. Supplementary Table 1 describes the full search term used in each database searched.

### Eligibility criteria

The inclusion criteria for our systematic review and meta-analysis were as follows: (1) Randomized or observational studies, (2) that performed a direct comparison between aSAH patients treated with or without milrinone in adult patients, (3) reporting of baseline patients characteristics, (4) reporting presence of delayed cerebral ischemia related to vasospasm or clinical outcome at 3–6 months. Conference abstracts, single-arm studies, case reports, animal studies were excluded. Two investigators (KG and AA) independently screened and selected the studies for the final review. Discrepancies were resolved by a third investigator (AS).

### Data extraction

Extracted data included study design, country and year of the study, baseline characteristics, primary, and secondary outcomes. Two investigators (KG and NA) independently extracted the data from the included studies. Any disagreement was resolved by consensus.

### Risk of bias assessment and quality of evidence

The methodological quality of the observational studies was evaluated using the Newcastle Ottawa scale (NOS) (6). Observational studies with total scores of ≥ 6 on NOS were considered to have a low risk of bias (Supplementary Table 2a). Clinical trials were assessed using the Jadad composite scale (7), which incorporates randomization, blinding, and withdrawals, with scores ranging from 0 to 5; a cumulative score of ≥3 was considered to represent a low risk of bias (Supplementary Table 2b). Funnel plot analysis was used to evaluate publication bias. The funnel plots for the mortality and good clinical outcome appeared asymmetric by visual inspection (Supplementary Figures 1, 2). However, Egger’s regression analysis did not show evidence of publication bias. It should be noted, however, that funnel plot interpretation is limited when the number of included studies is small, as in our meta-analysis. Therefore, while the quantitative assessment suggests lower risk, we cannot completely exclude the possibility of publication bias.

### Effect measures

The primary outcome of the analysis was the occurrence of delayed cerebral ischemia related to vasospasm. Secondary outcomes included good functional outcome, defined as a modified Rankin Scale (mRS) score of 0–2 at 3–6 months, and mortality at 3–6 months. Safety outcomes comprised cardiovascular adverse events.

### DCI definition

The definition of delayed cerebral ischemia was not uniform across the included studies. We therefore interpreted this endpoint as study defined DCI or vasospasm-related ischemic injury, rather than as a strictly consensus defined DCI outcome. The 2010 international consensus definition (8) recommends that DCI related outcome assessment should primarily include cerebral infarction identified on CT or MRI, after exclusion of procedure-related infarction. Clinical deterioration attributed to DCI may be used as a secondary outcome after exclusion of other potential causes.

Across the included studies, DCI definitions varied substantially. Labeyrie et al. (9) and Jentzsch et al. (13) defined DCI as cerebral infarction occurring within 3–21 days after ictus, excluding alternative causes such as iatrogenic injury, intracerebral hemorrhage, external ventricular drain placement, mechanical brain shift, herniation, or cardioembolism. Kotwal et al. (10) defined DCI as a new infarct on the last CT scan before discharge if unrelated to other causes. Koyanagi et al. (15) used a broader timeframe, defining DCI as newly developed cerebral infarction resulting from delayed ischemic neurologic deficits on CT or MRI within 6 weeks after subarachnoid hemorrhage. In contrast, Lakhal et al. (11) defined DCI more restrictively as ischemic sequelae on 6-month follow-up CT attributed to vasospasm.

This variability introduces clinical and methodological heterogeneity because the pooled DCI outcome combines radiographic infarction, delayed neurologic deterioration, and mixed clinical-radiographic definitions. In addition, vasospasm itself was assessed using different modalities and thresholds across studies, including TCD-based criteria such as elevated mean flow velocity or Lindegaard ratio, as well as CTA/DSA-based arterial narrowing. Therefore, the pooled DCI estimate should be interpreted cautiously and should not be assumed to represent a uniform consensus-defined DCI endpoint.

### Statistical analysis

Statistical analysis was performed using IBM SPSS Software Statistics (Version 29.0). Absolute counts are provided in addition to effect estimates, which are expressed as odds ratios (OR) with corresponding 95% confidence intervals (CI). The heterogeneity of the results was evaluated using the *I*^2^ statistic as defined by the Cochrane handbook for systematic reviews.

### Sensitivity analysis

To confirm the robustness of our results, sensitivity analysis for the good functional outcome, mortality and delayed cerebral ischemia using a leave-one-out meta-analysis was performed to see if it had a significant influence on the meta-analysis result (Supplementary Figure 3). This association showed a consistent trend toward reduction in delayed cerebral ischemia, although statistical significance was not maintained. This suggests that the beneficial effect of milrinone infusion on reducing delayed cerebral ischemia is robust in direction, even if the magnitude may be influenced by individual studies.

### Standard protocol approvals and registrations

This systematic review and meta-analysis was registered in PROSPERO under registration number CRD420251131643. The registered protocol is available at: https://www.crd.york.ac.uk/PROSPERO/view/CRD420251131643. All data used in the analyses were extracted from articles available in the public domain through indexed databases. No individual patient-level data were accessed, and therefore informed consent and institutional review board approval were not required. The datasets generated and analyzed during the current study are fully available from the corresponding author upon reasonable request.

## Results

### Study selection

**Figure 1:**
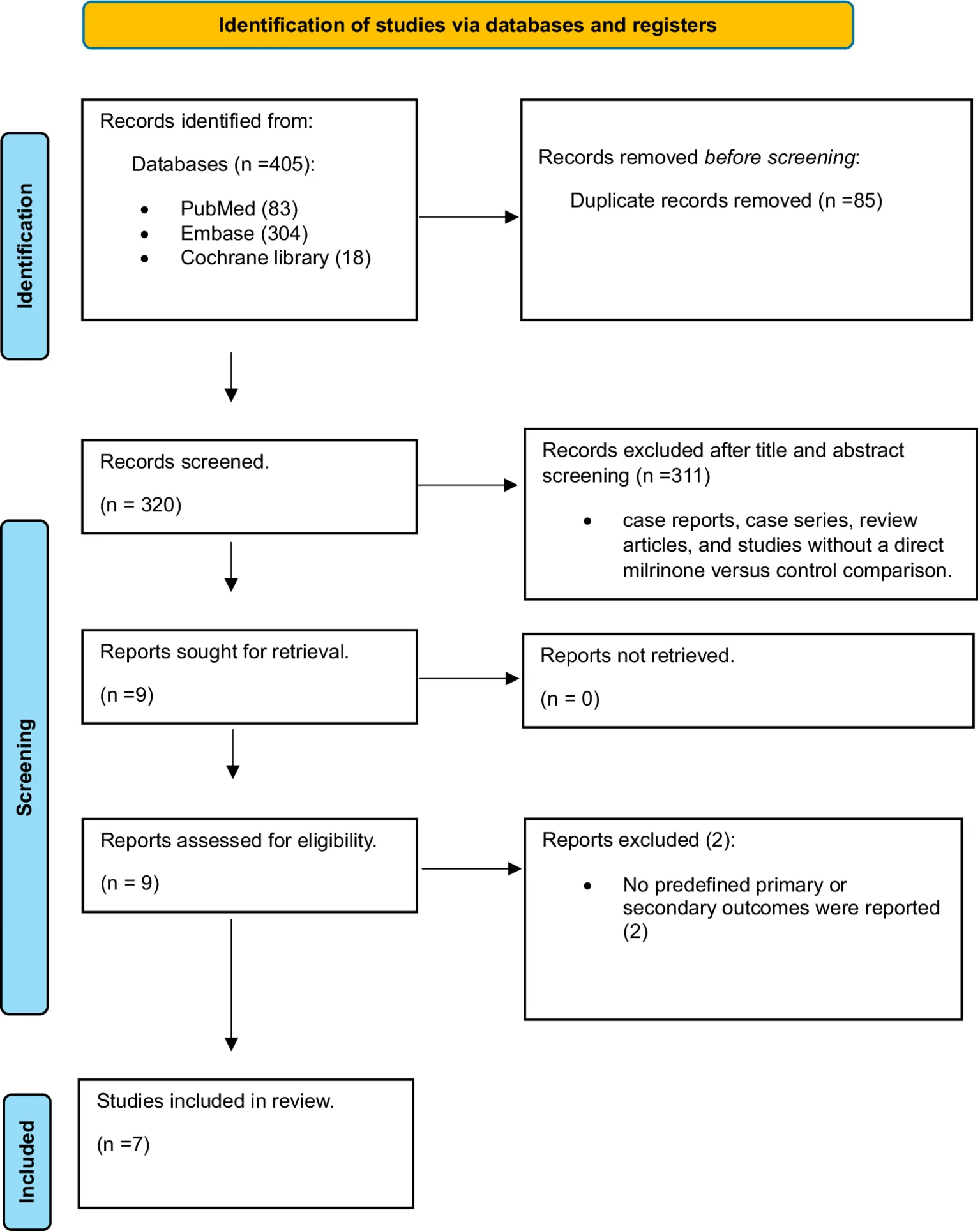
PRISMA flow diagram for the selection of studies.

A total of 405 studies were retrieved by our search strategy. Among these, 9 were eligible for the systematic review. Seven studies (9–14) met our inclusion criteria and were included in the meta-analysis, comprising a total of 1,045 patients, of whom 529 (50.6%) received milrinone infusion. Figure 1 shows the PRISMA flow chart illustrating how the final studies were selected. The basic characteristics of the included studies are summarized in Table 1. Although included studies generally demonstrated acceptable methodological quality by NOS/Jadad criteria, the overall body of evidence remains limited by small sample sizes, predominance of observational single-center studies, heterogeneous comparator groups, variable milrinone protocols, and potential residual confounding.

**Table 1:** Demographics and basic characteristics of the included studies.

Study	Region	Design	Site	Sample size	Gender	Treatment group	Control group	Fisher scale	Dose	Time of treatment initiation
					Male					
Koyanagi et al. (15)	Japan	Prospective	Single center	274	64 (23.4%)	Intrathecal milrinone + standard care	Standard Care	NR	0.87 mg for 2 h. Daily for 14 days	Within 24 h of surgical intervention
Soliman and Zohry (12)	Egypt	Randomized study	Single center	90	49 (54.4%)	IV milrinone + standard care	IV Magnesium + standard care	2, IQR 1	0.5 μg/kg/mi (for 24 h daily) without a loading dose for 21 days	Within 24 h of admission
Lakhal et al. (11)	France	Prospective	Single center	94	37 (39.4%)	IV milrinone + standard care	Standard care	4, IQR 1	0.5 μg/kg/min without a loading dose for 5 days	One hour after the attainment of a mean BP of 100–120 mmHg
Labeyrie et al. (9)	Europe	Retrospective	Multi-center	400	122 (30.5%)	IV milrinone + percutaneous balloon angioplasty + standard care	Standard care	4, IQR 1	1.5 μg/kg/min for 15 days, no loading dose	within 2 h from clinical or radiological suspicion of DCI,
Kotwal et al. (10)	India	Prospective	Single center	34	14 (41.2%)	IV milrinone + standard care	IA Nimodipine + standard care	3 IQR 2	0.3 μg/kg/min	Upon DCI detection
Jentzsch et al. (13)	Germany	Retrospective	Single center	23	9 (39.1%)	IA milrinone + standard care	IA Nimodipine + standard care	4, IQR 1	10–20 mg per vascular territory. Repeated on demand	NA
Baang et al. (14)	USA	Retrospective	Single center	130	43 (33.1%)	IV milrinone + standard care	Standard care	NR	bolus of 50 μg/kg followed by an infusion at a rate of 0.5–1.25 μg/kg/min. 7 days	Upon DCI detection on TCD, CTA or DSA

NR, not reported.

Standard care consisted of oral nimodipine, maintenance of euvolemia, and blood pressure augmentation with intravenous fluid resuscitation as clinically indicated.

### Primary analysis: intravenous milrinone only

#### Efficacy outcomes

##### Delayed cerebral ischemia related to vasospasm

**Figure 2:**
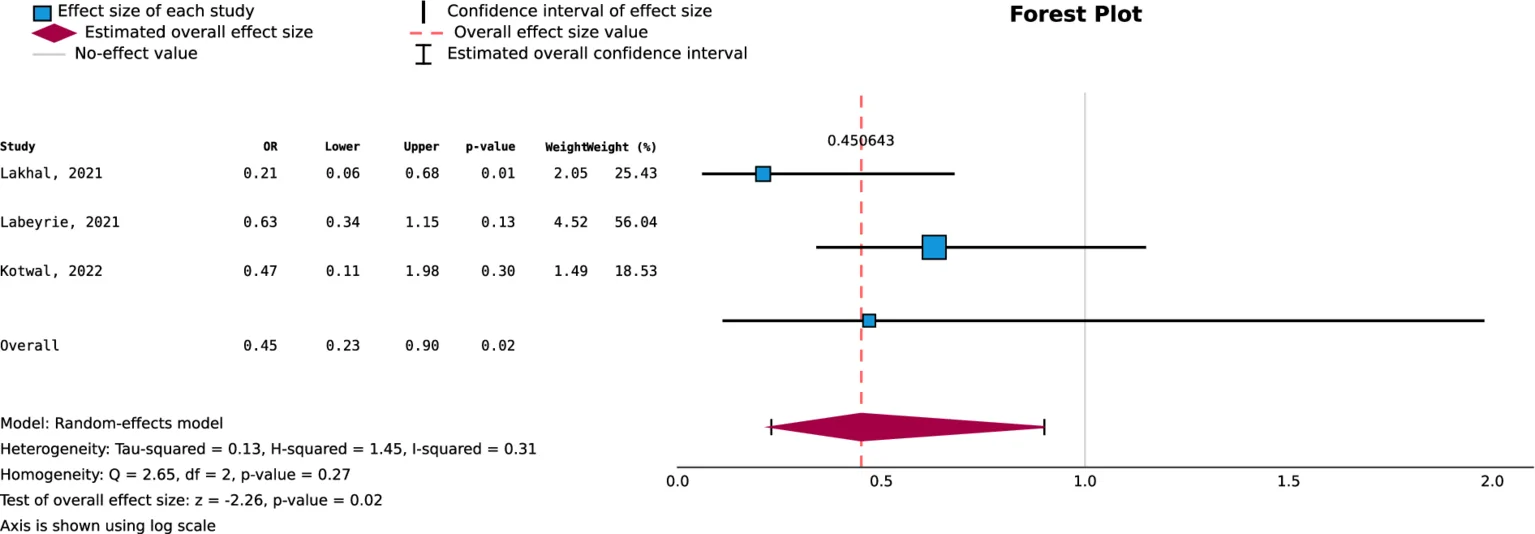
Forest plot showing the association of IV Milrinone infusion and DCI in patients with SAH.

Rates of delayed cerebral ischemia related to vasospasm were reported across three intravenous milrinone studies. In the intravenous milrinone group, DCI occurred in 31 of 254 patients (12.2%), compared with 63 of 274 patients (23.0%) in the control group. Intravenous milrinone was associated with a significantly lower odds of DCI related to vasospasm, with a pooled OR of 0.45 (95% CI, 0.23–0.90; *p* = 0.02). Moderate heterogeneity was observed across studies (*I*^2^ = 31%) (Figure 2).

##### Functional independence at 3–6 months

**Figure 3:**
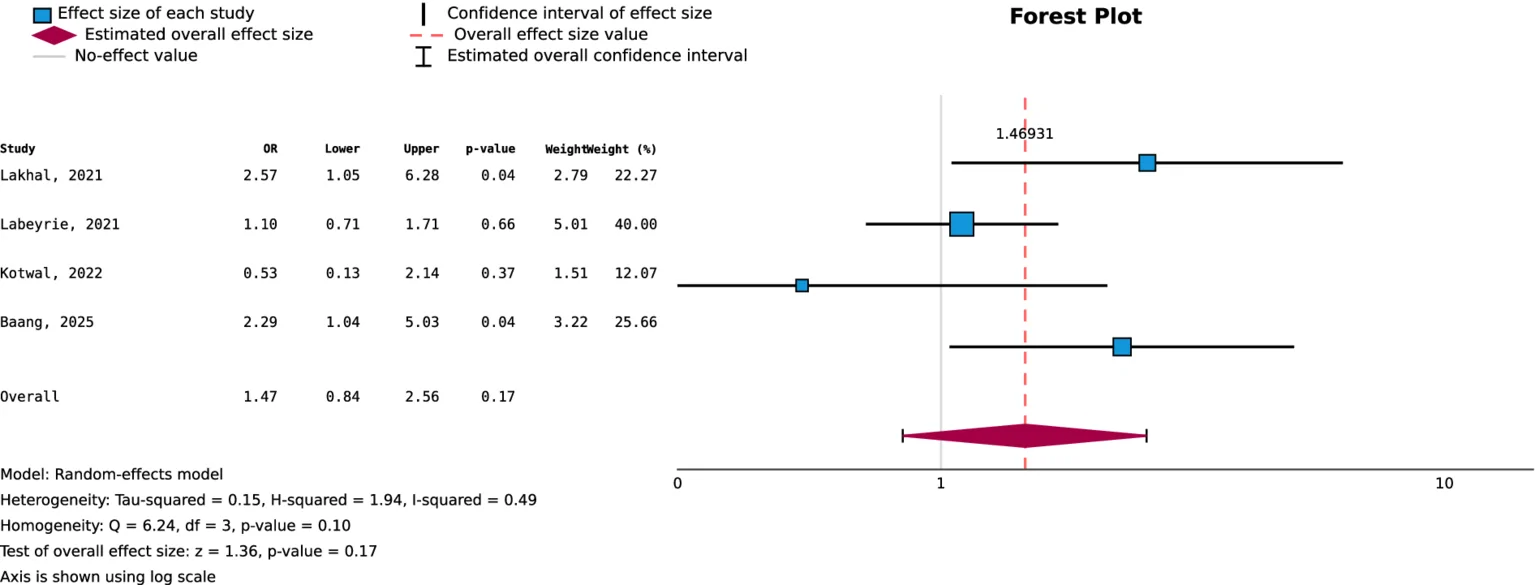
Forest plot showing the association of IV Milrinone infusion and good functional outcome in patients with SAH.

Functional independence, defined as modified Rankin Scale score 0–2 at 3–6 months, was reported in four intravenous milrinone studies, including 635 patients. Functional independence was achieved in 223 of 313 patients (71.2%) in the intravenous milrinone group and 207 of 322 patients (64.3%) in the control group. There was no statistically significant difference between groups, with a pooled OR of 1.47 (95% CI, 0.84–2.56; *p* = 0.17). Moderate heterogeneity was observed (*I*^2^ = 49%) (Figure 3).

#### Safety outcomes

##### All-cause mortality

**Figure 4:**
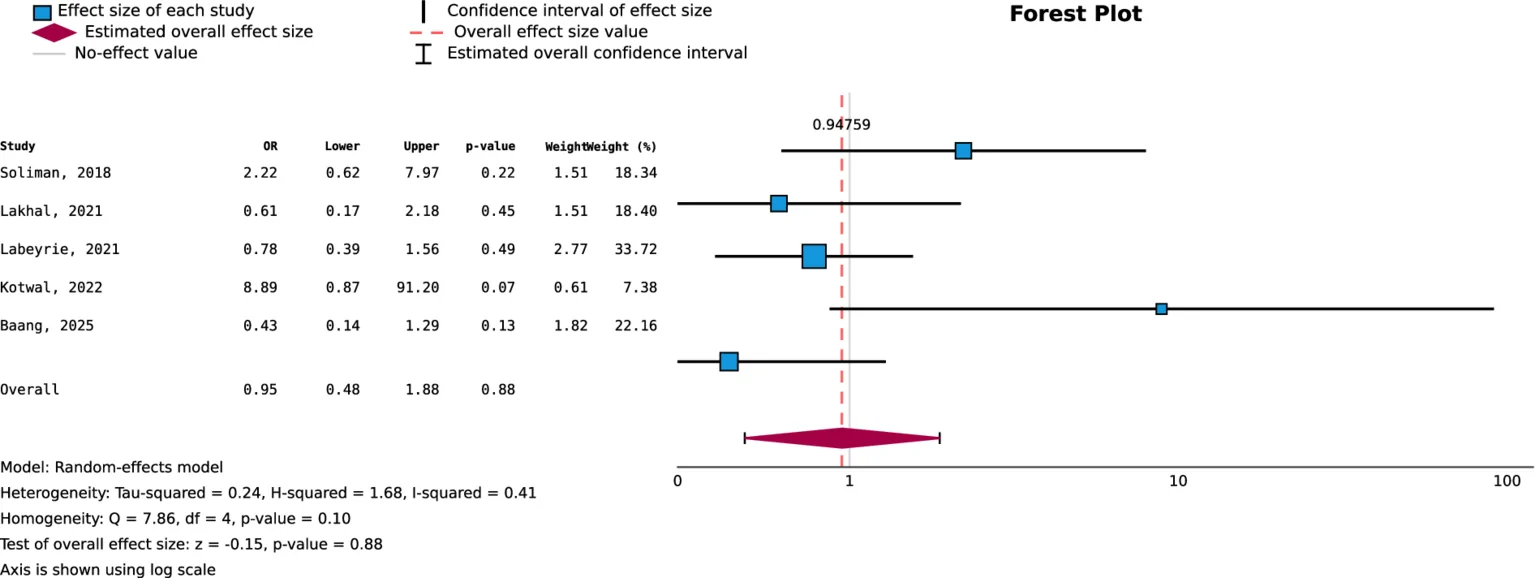
Forest plot showing the association of IV Milrinone infusion and mortality in patients with SAH.

All-cause mortality was reported in five intravenous milrinone studies. Mortality occurred in 38 of 358 patients (10.6%) in the intravenous milrinone group and 43 of 367 patients (11.7%) in the control group. There was no statistically significant difference in mortality between groups, with a pooled OR of 0.95 (95% CI, 0.48–1.88; *p* = 0.88). Moderate heterogeneity was observed (*I*^2^ = 41%) (Figure 4).

##### Cardiovascular adverse events

**Figure 5:**
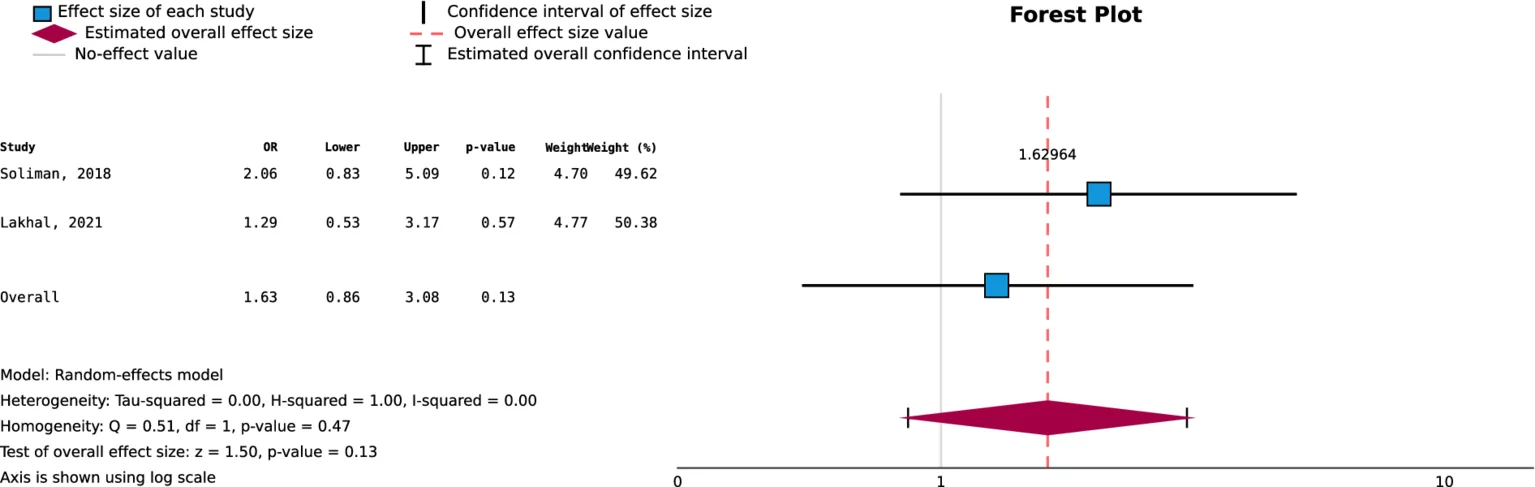
Forest plot showing the association of IV Milrinone infusion and cardiac adverse events in patients with SAH.

Cardiovascular adverse events, including hypotension, arrhythmia, or heart failure requiring vasopressors or antiarrhythmic therapy, were reported in two intravenous milrinone studies, including 184 patients. Cardiovascular adverse events occurred in 31 of 86 patients (36.0%) in the intravenous milrinone group and 25 of 98 patients (25.5%) in the control group. The pooled OR was 1.63 (95% CI, 0.86–3.08; *p* = 0.13), indicating no statistically significant difference between groups. No statistical heterogeneity was observed (*I*^2^ = 0%) (Figure 5).

### Secondary/exploratory analysis: all routes combined

#### Efficacy outcomes

##### Delayed cerebral ischemia related to vasospasm

**Figure 6:**
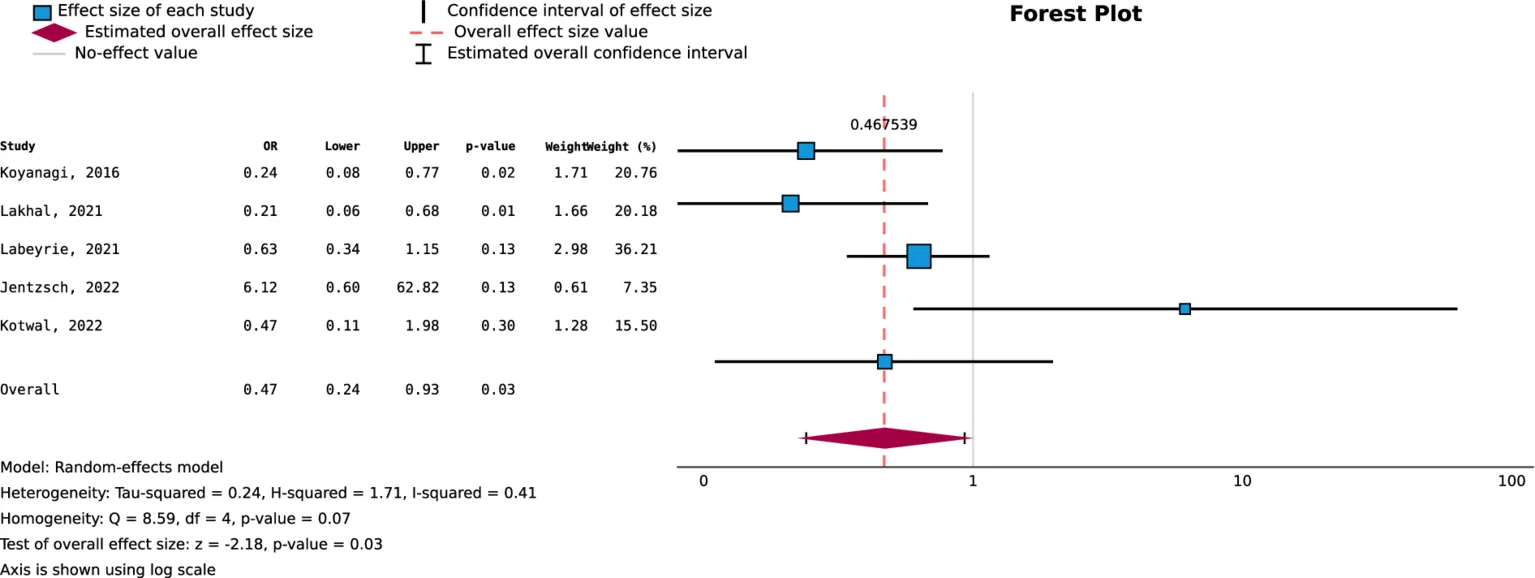
Forest plot showing the association of all route Milrinone infusion and DCI in patients with SAH.

Across five studies including all administration routes, DCI related to vasospasm occurred in 42 of 361 patients (11.6%) in the milrinone group and 85 of 384 patients (22.1%) in the control group. The pooled OR was 0.47 (95% CI, 0.24–0.93; *p* < 0.03), with moderate heterogeneity (*I*^2^ = 41%) (Figure 6).

##### Functional independence at 3–6 months

**Figure 7:**
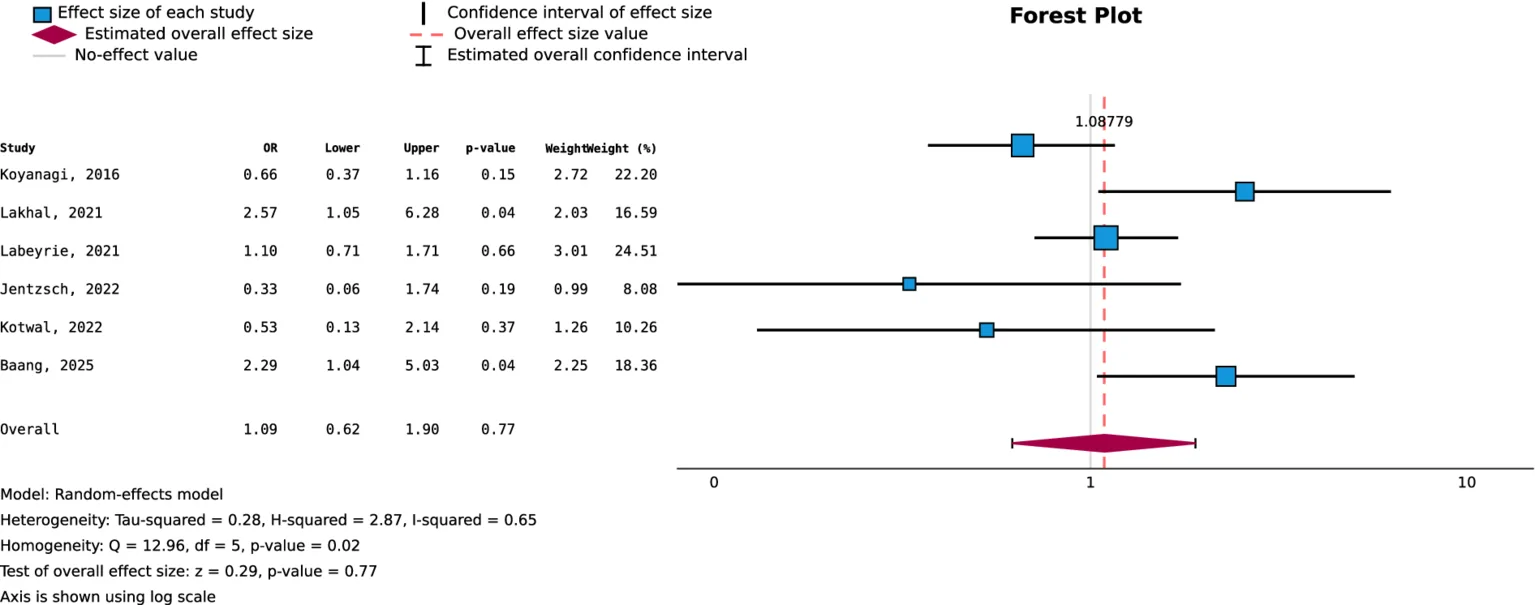
Forest plot showing the association of all route Milrinone infusion and good functional outcome in patients with SAH.

Functional independence was reported in six studies including 859 patients. Modified Rankin Scale score 0–2 at 3–6 months was achieved in 279 of 423 patients (65.9%) in the milrinone group and 278 of 436 patients (63.8%) in the control group. There was no statistically significant difference between groups, with a pooled OR of 1.09 (95% CI, 0.62–1.90; *p* = 0.77). Heterogeneity was high (*I*^2^ = 65%) (Figure 7).

### Safety outcomes

#### All-cause mortality

**Figure 8:**
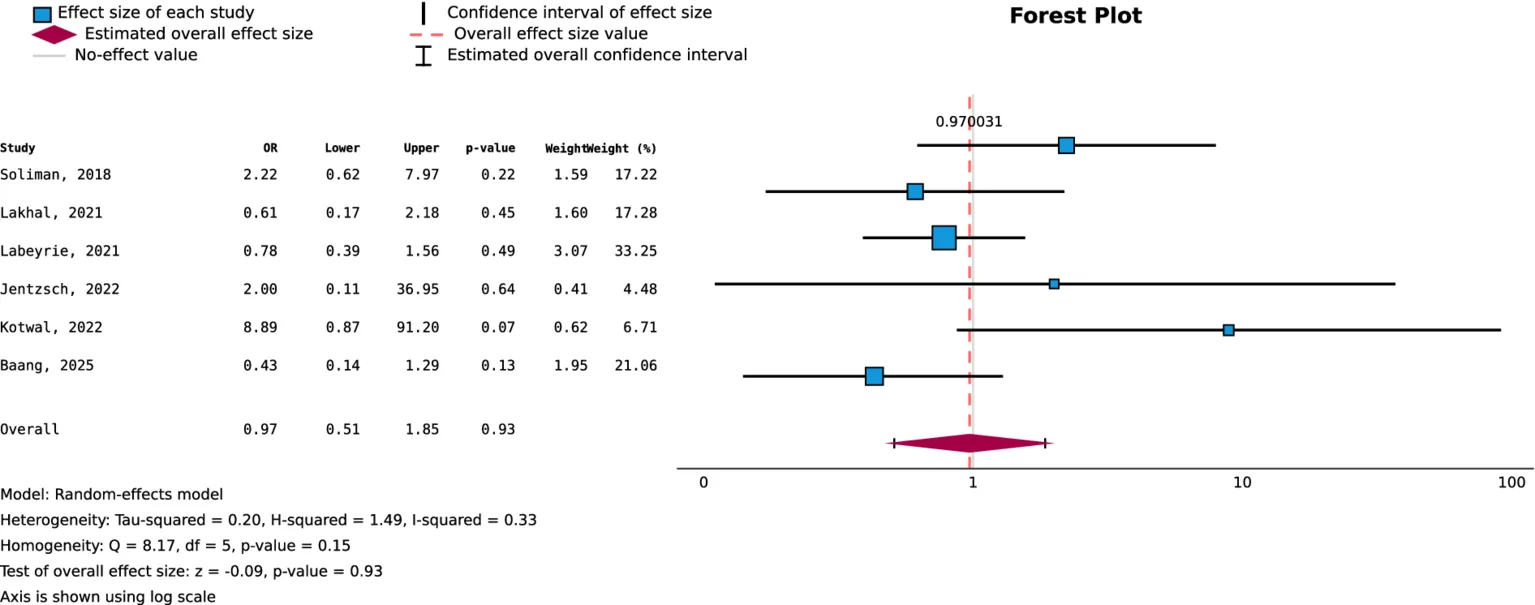
Forest plot showing the association of all route Milrinone infusion and mortality in patients with SAH.

Across six studies, mortality occurred in 39 of 366 patients (10.7%) in the milrinone group and 44 of 382 patients (11.5%) in the control group. The pooled OR was 0.97 (95% CI, 0.51–1.85; *p* = 0.93), suggesting no significant difference between groups. Moderate heterogeneity was observed (*I*^2^ = 33%) (Figure 8).

#### Cardiovascular adverse events

**Figure 9:**
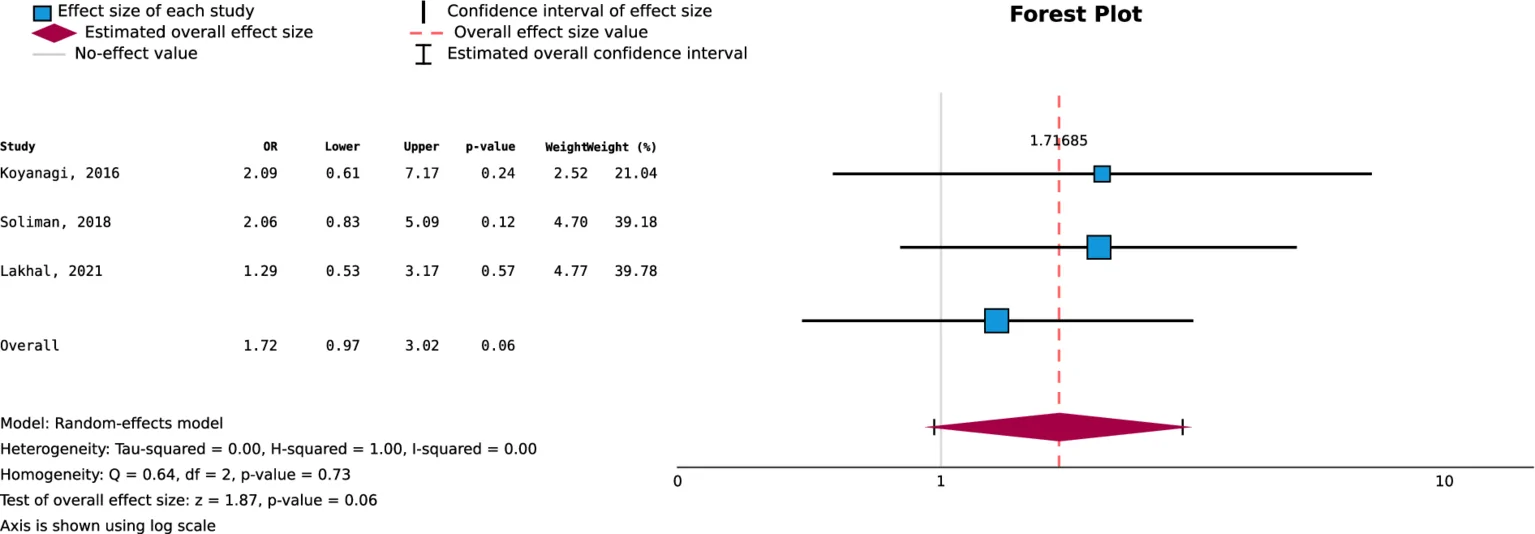
Forest plot showing the association of all route Milrinone infusion and cardiac adverse events in patients with SAH.

Cardiovascular adverse events were reported in three studies including 382 patients. Events occurred in 39 of 185 patients (21.1%) in the milrinone group and 29 of 197 patients (14.7%) in the control group. The pooled OR was 1.72 (95% CI, 0.97–3.02; *p* = 0.06). No statistical heterogeneity was observed (*I*^2^ = 0%) (Figure 9).

#### Comparator-specific subgroup analysis: IV milrinone versus standard care

In this subgroup, intravenous milrinone was associated with significantly higher odds of good functional outcome compared with standard care, defined as modified Rankin Scale score 0–2 at follow-up (OR = 2.41, 95% CI 1.33–4.34; *p* < 0.001). Mortality was numerically lower in the intravenous milrinone group; however, this difference did not reach statistical significance (OR = 0.50, 95% CI 0.22–1.14; *p* = 0.10).

## Discussion

Aneurysmal subarachnoid hemorrhage patients are at risk of developing cerebral artery vasospasm (CVS) that may lead to ischemic injury. Delayed cerebral ischemia is a term used to describe this brain injury (14). It is defined as a new focal neurological deficit or a decrease in the level of consciousness [reduction of at least 2 points in the Glasgow Coma Scale (GCS)] that lasts for at least 1 h and is not explained by other causes. It should also not be apparent immediately after aneurysm occlusion (16). DCI can occur in approximately 30% of aSAH patients between 3 and 14 days after the initial bleed, and it is associated with substantial morbidity and mortality (12). Among those who develop DCI, 15–20% will die or experience an ischemic stroke (4).

In this meta-analysis, we demonstrated that IV milrinone infusion was associated with a significantly lower incidence of DCI related to vasospasm [OR 0.45 (95% CI, 0.23–0.90; *p* = 0.02)]. However, this reduction in DCI did not translate into better functional outcomes [OR 1.47 (95% CI, 0.84–2.56; *p* = 0.17)] or reduced mortality [OR 0.95 (95% CI, 0.48–1.88; *p* = 0.88)]. Several explanations may account for this discrepancy. First, the available evidence remains underpowered for patient-centered outcomes such as mRS and mortality, which require larger sample sizes than radiographic or vasospasm-related endpoints. Second, DCI definitions varied across included studies and included radiographic infarction, delayed neurological deterioration, and mixed clinical-radiographic endpoints; therefore, the pooled DCI outcome may not fully capture the total burden of secondary brain injury after aSAH. Third, DCI is a multifactorial process involving endothelial injury with microspasm, blood–brain barrier disruption, microthrombosis, cortical spreading depolarizations, and impaired cerebral autoregulation, all of which predispose patients to secondary ischemic injury (13–18). Fourth, most included studies were observational and potentially affected by selection bias and survivor bias, confounding by disease severity, variation in rescue therapy, and differences in co-interventions. Finally, competing risks such as early brain injury, rebleeding, hydrocephalus, infection, cardiopulmonary complications, and withdrawal of care may dilute any downstream clinical benefit from reducing vasospasm-related ischemia.

This disconnect between vasospasm modification and clinical outcome is not unique to milrinone. Prior data on nimodipine show that it improves overall outcome after aneurysmal SAH, although nimodipine-only analyses did not demonstrate a statistically significant reduction in angiographically detected vasospasm (19). Conversely, clazosentan, an endothelin receptor antagonist, reduces angiographic vasospasm and vasospasm-related DCI, but randomized-trial meta-analysis data show no significant improvement in functional outcome or mortality (20). Together, these observations reinforce that vasospasm and DCI are important but incomplete surrogate endpoints.

Established predictors of DCI include large amounts of extravasated blood, poor clinical status on admission, smoking, hyperglycemia, hydrocephalus, diabetes mellitus, systemic inflammatory response syndrome, and a history of hypertension (17). DCI is typically detected using serial transcranial Doppler with assessment of cerebral blood flow velocity (CBFV) and Lindegaard ratios. A symptomatic cutoff of mean MCA CBFV ≥120 cm/s has been associated with a 57% risk of DCI (15). In deeply sedated or mechanically ventilated ICU patients, early clinical detection of neurological decline remains challenging, increasing reliance on CT perfusion imaging to identify regional hypoperfusion. Ultimately, infarctions occurring within 6 weeks of aSAH are the primary determinant of long-term outcomes and are associated with 30% higher healthcare costs (13).

Milrinone, a phosphodiesterase-3 inhibitor, increases intracellular cAMP and cGMP, resulting in improved inotropy, enhanced lusitropy, and smooth muscle relaxation within both arterial and venous vasculatures. These effects increase cardiac output, promote vasodilation, and may improve cerebral microcirculation (4). Preliminary data also suggest that milrinone may attenuate inflammatory cytokines such as IL-6 and TNF-α, which contribute to neuroinflammation and vasospasm (16). Milrinone has demonstrated effectiveness through intravenous administration and intra-arterial infusion (13).

Despite its reported benefits, it does come with side effects which we need to be aware of. It’s administration has been associated with increased cardiac output (CO), vasodilation and episodes of hypotension, occasionally necessitating administration of norepinephrine. It also can increase renal blood flow, and since aSAH and CVS are already associated with increased GFR and hyponatremia, it can result in further increased urine output and exacerbation of hyponatremia. Moreover, milrinone infusions can also result in cardiac rhythm disorders (21). In our study, cardiovascular adverse events were reported inconsistently across the included studies and variably included hypotension, arrhythmia, heart failure, vasopressor requirement, or antiarrhythmic therapy. In the primary IV-only analysis, cardiovascular events occurred in 31 of 86 patients (36.0%) in the milrinone group compared with 25 of 98 patients (25.5%) in the control group, with no statistically significant difference between groups (OR 1.63, 95% CI 0.86–3.08; *p* = 0.13). In the exploratory all-route analysis, events occurred in 39 of 185 patients (21.1%) receiving milrinone and 29 of 197 patients (14.7%) in the control group (OR 1.72, 95% CI 0.97–3.02; *p* = 0.06). Component level data for hypotension, arrhythmia, heart failure, and norepinephrine requirement were not uniformly reported, so separate pooled estimates could not be calculated. Importantly, the need for arterial pressure support may reflect either protocolized blood pressure augmentation or a true cardiovascular adverse event; therefore, this safety signal should be interpreted cautiously.

Administration route and therapeutic intent varied substantially across the included studies. Intravenous milrinone was generally administered systemically for the prevention or treatment of vasospasm-related DCI, whereas intra-arterial milrinone was used as an endovascular rescue therapy and intrathecal milrinone was delivered locally for prophylaxis. These approaches differ in pharmacokinetics, systemic exposure, procedural requirements, and adverse event profiles. Accordingly, the pooled analysis combining all administration routes should be interpreted as exploratory, while the intravenous only subgroup provides the most clinically coherent estimate of treatment effect. Evidence supporting intra-arterial and intrathecal administration remains limited and cannot be considered directly interchangeable with continuous intravenous infusion.

Lakhal et al. (11) reported that rescue endovascular angioplasty was required in 6 of 41 patients receiving milrinone (15%) compared with 28 of 53 controls (53%). However, this finding was derived from a single controlled before and after study, and rescue angioplasty was neither a prespecified outcome nor included in the quantitative meta-analysis because it was inconsistently reported across studies.

Our meta-analysis has several important limitations that warrant consideration. First, the number of available studies was relatively small, and several of the included observational studies enrolled only modest sample sizes, which may reduce the statistical power and generalizability of our findings. Second, the overall quality of evidence is limited, as only one randomized controlled trial was available, with the remainder consisting of observational, mostly single-center studies, which inherently carry a higher risk of bias and confounding. Third, we observed significant statistical heterogeneity for some outcomes, reflecting differences in study design, populations, and treatment protocols, which may have influenced the pooled estimates.

Another important limitation relates to variability in the comparator groups across studies. While some studies used standard medical management with nimodipine and blood pressure augmentation as the control, others compared milrinone against alternative therapies such as intravenous magnesium, intra-arterial nimodipine, or even endovascular angioplasty. This lack of a standardized control arm limits the ability to directly assess the relative efficacy of milrinone compared with a uniform standard of care. Additionally, differences in dosing regimens, administration routes, and outcome definitions (particularly for delayed cerebral ischemia). Additionally, cardiovascular adverse events were reported in only three studies, encompassing 382 of 1,045 patients (36.6%); therefore, the pooled safety estimate may be underpowered and may not fully represent the cardiovascular risk associated with milrinone. Finally, this study is an aggregate study level meta analysis; individual patient level data were not available, which limits the ability to adjust for patient-specific covariates or perform more granular subgroup analyses.

## Conclusion

In conclusion, intravenous milrinone may be associated with lower odds of DCI after aSAH, but this association was sensitive to the influence of individual studies and therefore remains uncertain. Cardiovascular adverse events were numerically increased in both the primary IV-only and exploratory all-route analyses, warranting cautious use and close hemodynamic monitoring. No significant improvement in functional outcome or mortality was demonstrated. These findings underscore the need for well-designed, adequately powered randomized controlled trials to confirm the efficacy and safety of milrinone and to clarify its impact on long-term clinical outcomes.

## Data Availability

The original contributions presented in the study are included in the article/Supplementary material, further inquiries can be directed to the corresponding author.
