# Supplementary material for: Efficacy and safety of milrinone infusion in patients with aneurysmal subarachnoid hemorrhage: a systematic review and meta-analysis
Source: Front Neurol. 2026 Sep 9;17:1872442. doi: 10.3389/fneur.2026.1872442 (PMC13597219; doi:10.3389/fneur.2026.1872442)
Supplement: Supplementary file 1 [file Supplementary_file_1.DOCX]

**Supplementary Table 1:** Search strategy used in each database searched.

| Database | Search Strategy | Articles retrieved |
| --- | --- | --- |
| MEDLINE | ("subarachnoid haemorrhage"[All Fields] OR "subarachnoid hemorrhage"[MeSH Terms] OR ("subarachnoid"[All Fields] AND "hemorrhage"[All Fields]) OR "subarachnoid hemorrhage"[All Fields] OR "sah"[All Fields]) AND ("vasospasm"[All Fields] OR "vasospasmic"[All Fields] OR "vasospasms"[All Fields] OR (("delay"[All Fields] OR "delayed"[All Fields] OR "delaying"[All Fields] OR "delays"[All Fields]) AND ("cerebral ischaemia"[All Fields] OR "cerebral infarction"[MeSH Terms] OR ("cerebral"[All Fields] AND "infarction"[All Fields]) OR "cerebral infarction"[All Fields] OR ("cerebral"[All Fields] AND "ischemia"[All Fields]) OR "cerebral ischemia"[All Fields] OR "brain ischemia"[MeSH Terms] OR ("brain"[All Fields] AND "ischemia"[All Fields]) OR "brain ischemia"[All Fields]))) AND ("milrinone"[Supplementary Concept] OR "milrinone"[All Fields] OR "milrinone"[MeSH Terms] OR "milrinone s"[All Fields]) | 83 |
| Embase | ('subarachnoid hemorrhage'/exp OR 'subarachnoid hemorrhage' OR sah) AND ('vasospasm' OR 'delayed cerebral ischemia') AND 'milrinone' | 304 |
| Cochrane CENTRAL | ((subarachnoid hemorrhage OR SAH) AND (vasospasm OR "delayed cerebral ischemia") AND milrinone):ti,ab,kw | 18 |

**Supplementary table 2.a:** Quality assessment of the included observational studies in the meta-analysis.

| Cohort studies | Selection | | | | Comparability | Outcome | | | Quality score |
| --- | --- | --- | --- | --- | --- | --- | --- | --- | --- |
|  | Representativeness of the exposed cohort | Selection of non-exposed cohort | Ascertainment of exposure | Demonstration that outcome of interest was not present at start of study | Comparability of the cohorts on the basis of design or analysis | Assessment of outcome | Was follow up long enough for outcomes to occur | Adequacy of follow up cohorts |  |
| Koyanagi, 2018 | 1 | 1 | 1 | 1 | 1 | 1 | 1 | 1 | 8 |
| Lakhal, 2021 | 0 | 1 | 1 | 1 | 1 | 1 | 1 | 1 | 7 |
| Labeyrie, 2021 | 1 | 1 | 1 | 1 | 1 | 1 | 1 | 1 | 8 |
| Jentzsch, 2022 | 1 | 1 | 1 | 1 | 0 | 1 | 1 | 1 | 7 |
| Kotwal ,2022 | 1 | 1 | 1 | 1 | 1 | 1 | 1 | 1 | 8 |
| Baang, 2025 | 1 | 1 | 1 | 1 | 1 | 1 | 1 | 1 | 8 |

**Supplementary Table 2.b:** Quality assessment of the included randomized studies in the meta-analysis.

| Randomized controlled studies | Randomization | | Blinding | | Withdrawals | Quality score |
| --- | --- | --- | --- | --- | --- | --- |
| Study | Was the study described as randomized? | Was the randomization appropriate? | Was the study described as double blind? | Was the double blinding appropriate? | Was there a description of withdrawals and dropouts? |  |
| Soliman,2018 ^9^ | 1 | 1 | 0 | 0 | 1 | 3 |

**Supplementary Figure 1**: Funnel plots showing publication bias analysis of studies that assessed mortality.

**Supplementary Figure 2**: Funnel plots showing publication bias analysis of studies that assessed good functional outcome.

**Supplementary Figure 3a:** Sensitivity analysis using the leave-one-out method for the association of Milrinone with good functional outcome

**Supplementary Figure 3b:** Sensitivity analysis using the leave-one-out method for the association of Milrinone mortality

**Supplementary Figure 3c:** Sensitivity analysis using the leave-one-out method for the association of Milrinone mortality
